# Chiropractic care and the risk of vertebrobasilar stroke: results of a case–control study in U.S. commercial and Medicare Advantage populations

**DOI:** 10.1186/s12998-015-0063-x

**Published:** 2015-06-16

**Authors:** Thomas M Kosloff, David Elton, Jiang Tao, Wade M Bannister

**Affiliations:** Optum Health – Clinical Programs at United Health Group, 11000 Optum Circle, Eden Prairie, MN 55344 USA; Optum Health – Clinical Analytics at United Health Group, 11000 Optum Circle, Eden Prairie, MN 55344 USA

**Keywords:** Chiropractic, Primary care, Cervical manipulation, Vertebrobasilar stroke, Adverse events

## Abstract

**Background:**

There is controversy surrounding the risk of manipulation, which is often used by chiropractors, with respect to its association with vertebrobasilar artery system (VBA) stroke. The objective of this study was to compare the associations between chiropractic care and VBA stroke with recent primary care physician (PCP) care and VBA stroke.

**Methods:**

The study design was a case–control study of commercially insured and Medicare Advantage (MA) health plan members in the U.S. population between January 1, 2011 and December 31, 2013. Administrative data were used to identify exposures to chiropractic and PCP care. Separate analyses using conditional logistic regression were conducted for the commercially insured and the MA populations. The analysis of the commercial population was further stratified by age (<45 years; ≥45 years). Odds ratios were calculated to measure associations for different hazard periods. A secondary descriptive analysis was conducted to determine the relevance of using chiropractic visits as a proxy for exposure to manipulative treatment.

**Results:**

There were a total of 1,829 VBA stroke cases (1,159 – commercial; 670 – MA). The findings showed no significant association between chiropractic visits and VBA stroke for either population or for samples stratified by age. In both commercial and MA populations, there was a significant association between PCP visits and VBA stroke incidence regardless of length of hazard period. The results were similar for age-stratified samples. The findings of the secondary analysis showed that chiropractic visits did not report the inclusion of manipulation in almost one third of stroke cases in the commercial population and in only 1 of 2 cases of the MA cohort.

**Conclusions:**

We found no significant association between exposure to chiropractic care and the risk of VBA stroke. We conclude that manipulation is an unlikely cause of VBA stroke. The positive association between PCP visits and VBA stroke is most likely due to patient decisions to seek care for the symptoms (headache and neck pain) of arterial dissection. We further conclude that using chiropractic visits as a measure of exposure to manipulation may result in unreliable estimates of the strength of association with the occurrence of VBA stroke.

## Background

The burden of neck pain and headache or migraine among adults in the United States is significant. Survey data indicate 13% of adults reported neck pain in the past 3 months [1]. In any given year, neck pain affects 30% to 50% of adults in the general population [2]. Prevalence rates were reportedly greater in more economically advantaged countries, such as the USA, with a higher incidence of neck pain noted in office and computer workers [3]. Similar to neck pain, the prevalence of headache is substantial. During any 3-month timeframe, severe headaches or migraines reportedly affect one in eight adults [1].

Neck pain is a very common reason for seeking health care services. “In 2004, 16.4 million patient visits or 1.5% of all health care visits to hospitals and physician offices, were for neck pain” [4]. Eighty percent (80%) of visits occurred as outpatient care in a physician’s office [4]. The utilization of health care resources for the treatment of headache is also significant. “In 2006, adults made nearly 11 million physician visits with a headache diagnosis, over 1 million outpatient hospital visits, 3.3 million emergency department visits, and 445 thousand inpatient hospitalizations” [1].

In the United States, chiropractic care is frequently utilized by individuals with neck and/or headache complaints. A national survey of chiropractors in 2003 reported that neck conditions and headache/facial pain accounted respectively for 18.7% and 12% of the patient chief complaints [5]. Chiropractors routinely employ spinal manipulative treatment (SMT) in the management of patients presenting with neck and/or headache [6], either alone or combined with other treatment approaches [7-10].

While evidence syntheses suggest the benefits of SMT for neck pain [7-9,11-13] and various types of headaches [10,12,14-16], the potential for rare but serious adverse events (AE) following cervical SMT is a concern for researchers [17,18], practitioners [19,20], professional organizations [21-23], policymakers [24,25] and the public [26,27]. In particular, the occurrence of stroke affecting the vertebrobasilar artery system (VBA stroke) has been associated with cervical manipulation. A recent publication [28] assessing the safety of chiropractic care reported, “…the frequency of serious adverse events varied between 5 strokes/100,000 manipulations to 1.46 serious adverse events/10,000,000 manipulations and 2.68 deaths/10,000,000 manipulations”. These estimates were, however, derived from retrospective anecdotal reports and liability claims data, and do not permit confident conclusions about the actual frequency of neurological complications following spinal manipulation.

Several systematic reviews investigating the association between stroke and chiropractic cervical manipulation have reported the data are insufficient to produce definitive conclusions about its safety [28-31]. Two case–control studies [32,33] used visits to a chiropractor as a proxy for SMT in their analyses of standardized health system databases for the population of Ontario (Canada). The more recent of these studies [32] also included a case-crossover methodology, which reduced the risk of bias from confounding variables. Both case–control studies reported an increased risk of VBA stroke in association with chiropractic visits for the population under age 45 years old. Cassidy, et al. [32] found, however, the association was similar to visits to a primary care physician (PCP). Consequently, the results of this study suggested the association between chiropractic care and stroke was noncausal. In contrast to these studies, which found a significant association between chiropractic visits and VBA stroke in younger patients (<45 yrs.), the analysis of a population-based case-series suggested that VBA stroke patients who consulted a chiropractor the year before their stroke were older (mean age 57.6 yrs.) than previously documented [34].

The work by Cassidy, et al. [32] has been qualitatively appraised as one of the most robustly designed investigations of the association between chiropractic manipulative treatment and VBA stroke [31]. To the best of our knowledge, this work has not been reproduced in the U.S. population. Thus, the main purpose of this study is to replicate the case–control epidemiological design published by Cassidy, et al. [32] to investigate the association between chiropractic care and VBA stroke; and compare it to the association between recent PCP care and VBA stroke in samples of the U.S. commercial and Medicare Advantage (MA) populations. A secondary aim of this study is to assess the utility of employing chiropractic visits as a proxy measure for exposure to spinal manipulation.

## Methods

### Study design and population

We developed a case–control study based on the experience of commercially insured and MA health plan members between January 1, 2011 and December 31, 2013. General criteria for membership in a commercial or MA health plan included either residing or working in a region where health care coverage was offered by the insurer. Individuals must have Medicare Part A and Part B to join a MA plan. The data set included health plan members located in 49 of 50 states. North Dakota was the only State not represented.

Both case and control data were extracted from the same source population, which encompassed national health plan data for 35,726,224 unique commercial and 3,188,825 unique MA members. Since members might be enrolled for more than one year, the average annual commercial membership was 14.7 million members and the average annual MA membership was 1.4 million members over the three year study period, which is comparable to ~5% of the total US population based on the data available from US Census Bureau [35]. Administrative claims data were used to identify cases, as well as patient characteristics and health service utilization.

The stroke cases included all patients admitted to an acute care hospital with vertebrobasilar (VBA) occlusion and stenosis strokes as defined by ICD-9 codes of 433.0, 433.01, 433.20, and 433.21 during the study period. Patients with more than one admission for a VBA stroke were excluded from the study. For each stroke case, four age and gender matched controls were randomly selected from sampled qualified members. Both cases and controls were randomly sorted prior to the matching using a greedy matching algorithm [36].

### Exposures

The index date was defined as the date of admission for the VBA stroke. Any encounters with a chiropractor or a primary care physician (PCP) prior to the index date were considered as exposures. To evaluate the impact of chiropractic and PCP treatment, the designated hazard period in this study was zero to 30 days prior to the index date. For the PCP analysis, the index date was excluded from the hazard period since patients might consult PCPs after having a stroke. The standard health plan coverage included a limit of 20 chiropractic visits. In rare circumstances a small employer may have selected a 12-visit limit. An internal analysis (data not shown) revealed that 5% of the combined (commercial and MA) populations reached their chiropractic visit limits. Instances of an employer not covering chiropractic care were estimated to be so rare that it would have had no measureable impact on the analysis. There were no limits on the number of reimbursed PCP visits per year.

### Analyses

Two sets of similar analyses were performed, one for the commercially insured population and one for the MA population. In each set of analyses, conditional logistic regression models were used to examine the association between the exposures and VBA strokes. To measure the association, we estimated the odds ratio of having the VBA stroke and the effect of total number of chiropractic visits and PCP visits within the hazard period. The analyses were applied to different hazard periods, including one day, three days, seven days, 14 days and 30 days for both chiropractic and PCP visits. The results of the chiropractic and PCP visit analyses were then compared to find evidence of excess risk of having stroke for patients with chiropractic visits during the hazard period. Previous research has indicated that most patients who experience a vertebral artery dissection are under the age of 45. Therefore, in order to investigate the impact of exposure on the population at different ages, separate analyses were performed on patients stratified by age (under 45 years and 45 years and up) for the study of the commercial population. The number of visits within the hazard period was entered as a continuous variable in the logistic model. The chi square test was used to analyze the proportion of co-morbidities in cases as compared to controls.

A secondary analysis was performed to evaluate the relevance of using chiropractic visits as a proxy for spinal manipulation. The commercial and MA databases were queried to identify the proportions of cases of VBA stroke and matched controls for which at least one chiropractic spinal manipulative treatment procedural code (CPT 98940 – 98942) was or was not recorded. The analysis also calculated the use of another manual therapy code (CPT 97140), which may be employed by chiropractors as an alternative means of reporting spinal manipulation.

### Ethics

The New England Institutional Review Board (NEIRB) determined that this study was exempt from ethics review.

## Results

The commercial study sample included 1,159 VBA stroke cases over the three year period and 4,633 age and gender matched controls. The average age of the patients was 65.1 years and 64.8% of the patients were male (Table 1). The prevalence rate of VBA stroke in the commercial population was 0.0032%.

**Table 1:** **Age and gender of cases and controls (Commercial)**

**Variable**	**Cases (n = 1159)**	**Controls (n = 4633)**
Age: mean (median)	65.1 (64.7)	65.1 (64.7)
Males: n (%)	751 (64.8)	3001 (64.8)

There were a total of 670 stroke cases and 2,680 matched controls included in the MA study. The average patient age was 76.1 years and 58.6% of the patients were male (Table 2). For the MA population, the prevalence rate of VBA stroke was 0.021%.

**Table 2:** **Age and gender of cases and controls (Medicare)**

**Variable**	**Cases (n = 670)**	**Controls (n = 2680)**
Age: mean (median)	76.1 (76.2)	76.1 (76.2)
Males: n (%)	393 (58.6)	1572 (58.6)

Claims during a one year period prior to the index date were extracted to identify comorbid disorders. Both the commercial and MA cases had a high percentage of comorbidities, with 71.5% of cases in the commercial study and 88.5% of the cases in the MA study reporting at least one of the comorbid conditions (Table 3). Six comorbid conditions of particular interest were identified, including hypertensive disease (ICD-9 401–404), ischemic heart disease (ICD-9 410–414), disease of pulmonary circulation (ICD-9 415–417), other forms of heart disease (ICD-9 420–429), pure hypercholesterolemia (ICD-9 272.0) and diseases of other endocrine glands (ICD-9 249–250). There were statistically significant differences (p = <0.05) between groups for most comorbidities. Greater proportions of comorbid disorders (p = <0.0001) were reported in the commercial and MA cases for hypertensive disease, heart disease and endocrine disorders (Table 3). The commercial cases also showed a larger proportion of diseases of pulmonary circulation, which was statistically significant (p = 0.0008). There were no significance differences in pure hypercholesterolemia for either the commercial or MA populations. Overall, cases in both the commercial and MA populations were more likely (p = <0.0001) to have at least one comorbid condition.

**Table 3:** **Comorbid conditions**

**Conditions n (%)**	**Commercial**			**Medicare**		
	**Cases (n = 1159)**	**Controls (n = 4633)**	**p-value**	**Cases (n = 670)**	**Controls (n = 2680)**	**p-value**
Hypertensive disease	767 (66.2)	2078 (44.9)	<0.0001	554 (82.7)	1721 (64.2)	<0.0001
Ischemic heart disease	300 (25.9)	638 (13.8)	<0.0001	258 (38.5 )	563 (21.0)	<0.0001
Diseases of pulmonary circulation	29 (2.5)	55 (1.2)	0.0008	18 (2.7)	70 (2.6)	0.9140
Other forms of heart disease	357 (30.8)	800 (17.3)	<0.0001	306 (45.7)	713 (26.6)	<0.0001
Pure Hypercholesterolemia	9 (0.8)	24 (0.5)	0.2957	6 (0.9)	26 (1.0)	0.8590
Diseases of other endocrine glands	319 (27.5)	754 (16.3)	<0.0001	285 (42.5)	740 (27.6)	<0.0001
At least one of the conditions	829 (71.5)	2317 (50.0)	<0.0001	593 (88.5)	1885 (70.3)	<0.0001

Among the commercially insured, 1.6% of stroke cases had visited chiropractors within 30 days of being admitted to the hospital, as compared to 1.3% of controls visiting chiropractors within 30 days prior to their index date. Of the stroke cases, 18.9% had visited a PCP within 30 days prior to their index date, while only 6.8% of controls had visited a PCP (Table 4). The proportion of exposures for chiropractic visits was lower in the MA sample within the 30-day hazard period (cases = 0.3%; controls = 0.9%). However, the proportion of exposures for PCP visits was higher, with 21.3% of cases having PCP visits as compared to12.9% for controls (Table 5).

**Table 4:** **Chiropractic and PCP visits prior to the index date (Commercial)**

**Exposures**	**All**		**Age <45 yr**		**Age ≥45 yr**	
	**Cases (n = 1159)**	**Controls (n = 4633)**	**Cases (n = 98)**	**Controls (n = 392)**	**Cases (n = 1061)**	**Controls (n = 4241)**
Most recent DC Visit						
0-1 day: n (%)	3 (0.3)	11 (0.2)	*	*	3 (0.3)	11 (0.3)
0-3 days: n (%)	6 (0.5)	21 (0.5)	*	1 (0.3)	6 (0.6)	20 (0.5)
0-7 days: n (%)	8 (0.7)	31 (0.7)	*	1 (0.3)	8 (0.8)	30 (0.7)
0-14 days: n (%)	9 (0.8)	44 (0.9)	*	3 (0.8)	9 (0.8)	41 (1.0)
0-30 days: n (%)	19 (1.6)	62 (1.3)	2 (2.0)	7 (1.8)	17 (1.6)	55 (1.3)
Most recent PCP Visit						
1-1 day: n (%)	41 (3.5)	15 (0.3)	4 (4.1)	1 (0.3)	37 (3.5)	14 (0.3)
1-3 days: n (%)	78 (6.7)	41 (0.9)	8 (8.2)	2 (0.5)	70 (6.6)	39 (0.9)
1-7 days: n (%)	115 (9.9)	93 (2.0)	10 (10.2)	4 (1.0)	105 (9.9)	89 (2.1)
1-14 days: n (%)	157 (13.5)	165 (3.6)	12 (12.2)	15 (3.8)	145 (13.7)	150 (3.5)
1-30 days: n (%)	219 (18.9)	316 (6.8)	23 (23.5)	29 (7.4)	196 (18.5)	287 (6.8)

*Insufficient data to compute an estimate.

**Table 5:** **Chiropractic and PCP visits prior the index date (Medicare)**

**Exposures**	**Cases (n = 670)**	**Controls (n = 2680)**
Most recent DC Visit		
0-1 day: n (%)	*	4 (0.1)
0-3 days: n (%)	*	8 (0.3)
0-7 days: n (%)	*	9 (0.3)
0-14 days: n (%)	1 (0.1)	15 (0.6)
0-30 days: n (%)	2 (0.3)	24 (0.9)
Most recent PCP Visit		
1-1 day: n (%)	16(2.4)	18 (0.7)
1-3 days: n (%)	30 (4.5)	36 (1.3)
1-7 days: n (%)	55 (8.2)	97 (3.6)
1-14 days: n (%)	90 (13.4)	183 (6.8)
1-30 days: n (%)	143 (21.3)	346 (12.9)

*Insufficient data to compute an estimate.

The results from the analyses of both the commercial population and the MA population were similar (Tables 6, 7 and 8). There was no association between chiropractic visits and VBA stroke found for the overall sample, or for samples stratified by age. No estimated odds ratio was significant at the 95% confidence level. MA data were insufficient to calculate statistical measures of association for hazard periods less than 0–14 days for chiropractic visits. When stratified by age, the data were too sparse to calculate measures of association for hazard periods less than 0–30 days in the commercial population. The data were too few to analyze associative risk by headache and/or neck pain diagnoses (data not shown).

**Table 6:** **Estimated odds ratios and 95% confidence interval (Commercial)**

**Exposures**	**All**		**Age < 45 yr**		**Age > =45 yr**	
	**Odds ratio**	**95% CI**	**Odds ratio**	**95% CI**	**Odds ratio**	**95% CI**
Any DC Visit						
0-1 day	1.09	0.30-3.91	*	*	1.09	0.30-3.91
0-3 days	1.14	0.46-2.83	*	*	1.20	0.48-2.30
0-7 days	1.03	0.48-2.25	*	*	1.07	0.49-2.33
0-14 days	0.82	0.40-1.68	*	*	0.88	0.43-1.81
0-30 days	1.23	0.73-2.06	1.14	0.24-5.50	1.24	0.72-2.14
Any PCP Visit						
1-1 day	11.56	6.30-21.21	16.00	1.79-143.2	11.22	5.96-21.11
1-3 days	7.75	5.29-11.35	16.00	3.40-75.35	7.31	4.93-10.86
1-7 days	5.23	3.95-6.93	10.00	3.14-31.88	5.00	3.73-6.68
1-14 days	4.24	3.36-5.35	3.72	1.62-8.53	4.29	3.37-5.46
1-30 days	3.22	2.66-3.89	4.08	2.17-7.68	3.14	2.58-3.83

*Insufficient data to compute an estimate.

**Table 7:** **Estimated odds ratios and 95% CI (Medicare)**

**Exposures**	**Odds ratio**	**95% CI**
Any DC Visit		
0-1 day	*	*
0-3 days	*	*
0-7 days	*	*
0-14 days	0.26	0.03-2.00
0-30 days	0.32	0.08-1.39
Any PCP Visit		
1-1 day	3.66	1.85-7.26
1-3 days	3.38	2.07-5.51
1-7 days	2.37	1.68-3.34
1-14 days	2.09	1.60-2.73
1-30 days	1.81	1.46-2.25

*Insufficient data to compute an estimate.

**Table 8:** **Odds ratio and 95% CI for association between # of exposures during 30-day hazard period**

**Exposures**	**All cases**		**Age <45 yr**		**Age >45 yr**	
	**Odds ratio**	**95% CI**	**Odds ratio**	**95% CI**	**Odds ratio**	**95% CI**
Commercial						
Any DC* visit	1.03	0.86-1.26	1.32	0.64-2.71	1.01	0.81-1.25
Any PCP visit	2.01	1.77-2.29	2.38	1.55-3.66	1.97	1.72-2.26
Medicare						
Any DC* visit	0.54	0.23-1.28				
Any PCP visit	1.51	1.32-1.73				

*DC = Chiropractic.

These results showed there is an association existing between PCP visits and VBA stroke incidence regardless of age or length of hazard period. A strong association was found for those visits close to the index date (OR 11.56; 95% CI 6.32-21.21) for all patients with a PCP visit within 0–1 day hazard period in the commercial sample. There was an increased risk of VBA stroke associated with each PCP visit within 30-days prior to the index date for MA patients (OR 1.51; 95% CI 1.32-1.73) and commercial patients (OR 2.01; 95% CI 1.77-2.29).

The findings of the secondary analysis showed – that of 1159 stroke cases from commercial population – there were a total of 19 stroke cases associated with chiropractic visits for which 13 (68%) had claims documentation indicating chiropractic SMT was performed. For the control group of the commercial cohort, 62 of 4633 controls had claims of any kind of chiropractic visits and 47 of 4633 controls had claims of SMT. In the commercial control group, 47 of 62 DC visits (76%) included SMT in the claims data. Only 1 of 2 stroke cases in the MA population included SMT in the claims data. For the MA cohort, 21 of 24 control chiropractic visits (88%) included SMT in the claims data (Table 9).

**Table 9:** **Chiropractic (DC) visits with spinal manipulative treatment (SMT)**

	**Commercial**			**Medicare**		
	**DC visit with SMT**	**Any DC visit**	**Total # in sample**	**DC visit with SMT**	**Any DC visit**	**Total # in sample**
Stroke cases	13	19	1159	1	2	670
Controls	47	62	4633	21	24	2680
All	60	81	5792	22	26	3350

None of the stroke cases in either population included CPT 97140 as a substitute for the more conventionally reported chiropractic manipulative treatment procedural codes (98940 – 98942). For the control groups, there were three instances where CPT 97140 was reported without CPT 98940 – 98942 in the commercial population. The CPT code 97140 was not reported in MA control cohort.

## Discussion

The primary aim of the present study was to investigate the association between chiropractic manipulative treatment and VBA stroke in a sample of the U.S. population. This study was modelled after a case–control design previously conducted for a Canadian population [32]. Administrative data for enrollees in a large national health care insurer were analyzed to explore the occurrence of VBA stroke across different time periods of exposure to chiropractic care in comparison with PCP care.

Unlike Cassidy et al. [32] and most other case–control studies [33,37,38], our results showed there was no significant association between VBA stroke and chiropractic visits. This was the case for both the commercial and MA populations. In contrast to two earlier case–control studies [32,33], this lack of association was found to be irrespective of age. Although, our results (Table 8) did lend credence to previous reports that VBA stroke occurs more frequently in patients under the age of 45 years. Additionally, the results from the present study did not identify a relevant temporal impact. There was no significant association, when the data were sufficient to calculate estimates, between chiropractic visits and stroke regardless of the hazard period (timing of most recent visit to a chiropractor and the occurrence of stroke).

There are several possible reasons for the variation in results with previous similar case–control studies. The younger (<45 yrs.) commercial cohort that received chiropractic care in our study had noticeably fewer cases. The 0–30 days hazard period included only 2 VBA stroke cases. There were no stroke cases for other hazard periods in this population. In contrast, earlier studies reported sufficient cases to calculate risk estimates for most hazard periods [32,33].

Another factor that potentially influenced the difference in results concerns the accuracy of hospital claims data in the U.S. vs. Ontario, Canada. The source population in the Province of Ontario was identified, in part, from the Discharge Abstract Database (DAD). The DAD includes hospital discharge and emergency visit diagnoses that have undergone a standardized assessment by a medical records coder [39]. To the best of our knowledge, similar quality management practices were not routinely applied to hospital claims data used in sourcing the population for our study.

An additional reason for the disparity in results may be due to differences in the proportions of chiropractic visits where SMT was reportedly performed. Our study showed that SMT was not reported by chiropractors in more than 30% of commercial cases. It is plausible that a number of the cases in earlier studies also did not include SMT as an intervention. Differences between studies in the proportion of cases reporting SMT may have affected the calculation of risk estimates.

Also, there were an insufficient number of cases having cervical and/or headache diagnoses in our study. Therefore, our sample population may have included proportionally less cases where cervical manipulation was performed.

Our results were consistent with previous findings [32,33] in showing a significant association between PCP visits and VBA stroke. The odds ratios for any PCP visit increase dramatically from 1–30 days to 1–1 day (Tables 6 and 7). This finding is consistent with the hypothesis that patients are more likely to see a PCP for symptoms related to vertebral artery dissection closer to the index date of their actual stroke. Since it is unlikely that the services provided by PCPs cause VBA strokes, the association between recent PCP visits and VBA stroke is more likely attributable to the background risk related to the natural history of the condition [32].

A secondary goal of our study was to assess the utility of employing chiropractic visits as a surrogate for SMT. Our findings indicate there is a high risk of bias associated with using this approach, which likely overestimated the strength of association. Less than 70% of stroke cases (commercial and MA) associated with chiropractic care included SMT. A somewhat higher proportion of chiropractic visits included SMT for the control groups (commercial = 76%; MA = 88%).

There are plausible reasons that support these findings. Internal analyses of claims data (not shown) consistently demonstrate that one visit is the most common number associated with a chiropractic episode of care. The single visit may consist of an evaluation without treatment such as SMT. Further; SMT may have been viewed as contraindicated due to signs and symptoms of vertebral artery dissection (VAD) and/or stroke. This might explain the greater proportion of SMT provided to control groups in both the commercial and MA populations.

Overall, our results increase confidence in the findings of a previous study [32], which concluded there was no excess risk of VBA stroke associated chiropractic care compared to primary care. Further, our results indicate there is no significant risk of VBA stroke associated with chiropractic care. Additionally, our findings highlight the potential flaws in using a surrogate variable (chiropractic visits) to estimate the risk of VBA stroke in association with a specific intervention (manipulation).

Our study had a number of strengths and limitations. Both case and control data were extracted from the same source population, which encompassed national health plan data for approximately 36 million commercial and 3 million MA members. A total of 1,829 cases were identified, making this the largest case–control study to investigate the association between chiropractic manipulation and VBA stroke. Due to the nationwide setting and large sample size, our study likely reduced the risk of bias related to geographic factors. However, there was a risk of selection bias – owing to the data set being from a single health insurer – including income status, workforce participation, and links to health care providers and hospitals.

Our study closely followed a methodological approach that had previously been described [32], thus allowing for more confident comparisons.

The current investigation analyzed data for a number of comorbid conditions that have been identified as potentially modifiable risk factors for a first ischemic stroke [40]. The differences between groups were statistically significant for most comorbidities. Information was not obtainable about behavioural comorbid factors e.g., smoking and body mass. With the exception of hypertensive disease, there are reasons to question the clinical significance of these conditions in the occurrence of ischemic stroke due to vertebral artery dissection. A large multinational case-referent study investigated the association between vascular risk factors (history of vascular disease, hypertension, smoking, hypercholesterolemia, diabetes mellitus, and obesity/overweight) for ischemic stroke and the occurrence of cervical artery dissection [41]. Only hypertension had a positive association (odds ratio 1.67; 95% confidence interval, 1.32 to 2.1; P <0.0001) with cervical artery dissection.

While the effect of other unmeasured confounders cannot be discounted, there is reason to suspect the absence of these data was not deleterious to the results. Cassidy, et al. found no significant differences in the results their case-crossover design, which affords better control of unknown confounding variables, and the findings of their case–control study [32].

Our results highlight just how unusual VBA stroke is in the MA cohort (prevalence = 0.021%) and – even more so – for the commercial population (prevalence = 0.0032%). As a result, some limitations of this study related to the rarity of reporting VBA stroke events. Despite the larger number of cases, data were insufficient to calculate estimates and confidence intervals for seven measures of exposure (4 commercial and 3 MA) for chiropractic visits. Additionally, we were not able to compute estimates specifically for headache and neck pain diagnoses due to small numbers. Confidence intervals associated with estimates tended to be wide making the results imprecise [42].

There were limitations related to the use of administrative claims data. “Disadvantages of using secondary data for research purposes include: variations in coding from hospital to hospital or from department to department, errors in coding and incomplete coding, for example in the presence of comorbidities. Random errors in coding and registration of discharge diagnoses may dilute and attenuate estimates of statistical association” [43]. The recordings of unvalidated hospital discharge diagnostic codes for stroke have been shown to be less precise when compared to chart review [44,45] and validated patient registries [43,46]. Cassidy, et al. [32] conducted a sensitivity analysis to determine the effect of diagnostic misclassification bias. Their conclusions did not change when the effects of misclassification were assumed to be similarly distributed between chiropractic and PCP cases.

A particular limitation in using administrative claims data is the paucity of contextual information surrounding the clinical encounters between chiropractors/PCPs and their patients. Historical elements describing the occurrence/absence of recent trauma or activities reported in case studies [47-51] as potential risk factors for VBA stroke were not available in claims data. Confidence was low concerning the ability of claims data to provide accurate and complete reporting of other health disorders, which have been described in case–control designs as being associated with the occurrence of VBA stroke e.g., migraine [52] or recent infection [53]. Symptoms and physical examination findings that would have permitted further stratification of cases were not reported in the claims data.

The reporting of clinical procedures using current procedural terminology (CPT) codes presented additional shortcomings concerning the accuracy and interpretation of administrative data. One inherent constraint was the lack of anatomic specificity associated with the use of standardized procedural codes in claims data. Chiropractic manipulative treatment codes (CPT 98940 – 98942) have been formatted to describe the number of spinal regions receiving manipulation. They do not identify the particular spinal regions manipulated.

Also, treatment information describing the type(s) of manipulation was not available. When SMT was reported, claims data could not discriminate among the range of techniques including thrust or rotational manipulation, various non-thrust interventions e.g., mechanical instruments, soft tissue mobilizations, muscle energy techniques, manual cervical traction, etc. Many of these techniques do not incorporate the same biomechanical stressors associated with the type of manipulation (high velocity low amplitude) that has been investigated as a putative risk factor for VBA stroke [54-56]. It seems plausible that the utility of future VBA stroke research would benefit from explicit descriptions of the particular type of manipulation performed.

Moreover, patient responses to care – including any adverse events suggestive of vertebral artery dissection or stroke-like symptoms – were not obtainable in the data set used for the current study.

In the absence of performing comprehensive clinical chart audits, it is not possible to know from claims data what actually transpired in the clinical encounter. Further, chart notes may themselves be incomplete or otherwise fail to precisely describe the nature of interventions [57]. Therefore, manipulation codes represent surrogate measures, albeit more direct surrogate measures, than simply using the exposure to chiropractic visits.

Our study was also limited to replication of the case–control design described by Cassidy, et al. [32]. For pragmatic reasons, we did not attempt to conduct a case-crossover design. While the addition of a case-crossover design would have provided better control of confounding variables, Cassidy, et al. [32] showed the results were similar for both the case control and case crossover studies.

The findings of this case–control study and previous retrospective research underscore the need to rethink how to better conduct future investigations. Researchers should seek to avoid the use of surrogate measures or use the least indirect measures available. Instead, the focus should be on capturing data about the types of services and not the type of health care provider.

In alignment with this approach, it is also important for investigators to access contextual data (e.g., from electronic health records), which can be enabled by qualitative data analysis computer programs [58]. The acquisition of the elements of clinical encounters – including history, diagnosis, intervention, and adverse events – can provide the infrastructure for more actionable research. Because of the rarity of VBA stroke, large data sets (e.g., registries) containing these elements will be necessary to achieve adequate statistical power for making confident conclusions.

Until research efforts produce more definitive results, health care policy and clinical practice judgments are best informed by the evidence about the effectiveness of manipulation, plausible treatment options (including non-thrust manual techniques) and individual patient values [20].

## Conclusions

Our findings should be viewed in the context of the body of knowledge concerning the risk of VBA stroke. In contrast to several other case–control studies, we found no significant association between exposure to chiropractic care and the risk of VBA stroke. Our secondary analysis clearly showed that manipulation may or may not have been reported at every chiropractic visit. Therefore, the use of chiropractic visits as a proxy for manipulation may not be reliable. Our results add weight to the view that chiropractic care is an unlikely cause of VBA strokes. However, the current study does not exclude cervical manipulation as a possible cause or contributory factor in the occurrence of VBA stroke.
